# Metabolic programming of obesity by energy restriction during the perinatal period: different outcomes depending on gender and period, type and severity of restriction

**DOI:** 10.3389/fphys.2012.00436

**Published:** 2012-11-22

**Authors:** Catalina Picó, Mariona Palou, Teresa Priego, Juana Sánchez, Andreu Palou

**Affiliations:** Laboratory of Molecular Biology, Nutrition and Biotechnology (Nutrigenomics), University of the Balearic Islands, and CIBER de Fisiopatología de la Obesidad y NutriciónSpain

**Keywords:** calorie restriction, lactation, gestation, obesity, insulin and leptin sensitivity, developmental programming, hypothalamus, milk leptin

## Abstract

Epidemiological studies in humans and controlled intervention studies in animals have shown that nutritional programming in early periods of life is a phenomenon that affects metabolic and physiological functions throughout life. The phenotypes of health or disease are hence the result of the interaction between genetic and environmental factors, starting right from conception. In this sense, gestation and lactation are disclosed as critical periods. Continuous food restriction during these stages may lead to permanent adaptations with lasting effects on the metabolism of the offspring and may influence the propensity to develop different chronic diseases associated with obesity. However, the different outcomes of these adaptations on later health may depend on factors such as the type, duration, period, and severity of the exposure to energy restriction conditions, and they are, in part, gender specific. A better understanding of the factors and mechanisms involved in metabolic programming, and their effects, may contribute significantly to the prevention of obesity, which is considered to be one of the major health concerns of our time. Here, the different outcomes of maternal food restriction during gestation and lactation in the metabolic health of offspring, as well as potential mechanisms underlying these effects are reviewed.

## Introduction

The global epidemic of obesity, a multifactorial disease and key risk factor in the development of insulin resistance, type 2 diabetes, hypertension, and cardiovascular diseases is caused by both genetic and environmental factors. Its incidence has undergone a dramatic increase in recent decades resulting in a major impact on human morbidity, mortality, and quality of life, and becoming the main nutritional disorder in children and adolescents in developed countries (Friedman, 2003; Cripps et al., 2005). Overweight children have great risk of becoming overweight in adulthood (Von Kries et al., 1999); thus, the high prevalence of obesity together with the difficult success of its treatments makes it necessary to identify strategies to prevent obesity from the beginning of life. In this sense, there is a growing body of evidence suggesting that maternal nutritional conditions during critical stages of early life result in long term consequences on the future metabolic health of offspring, besides affecting their propensity to obesity in adulthood. In fact, both epidemiological studies in humans and intervention studies in animal models agree that the metabolic programming of energy balance begins with and can be modified by nutrition in the very early stages of development (Redman and Sweney, 1976; Lucas, 1991; Langley-Evans, 2006; Levin, 2006; Taylor and Poston, 2007; Wells, 2007; McMillen et al., 2008). Therefore, pregnancy and lactation are revealed as critical periods, where food restriction may lead to permanent adaptations with lasting effects on metabolic mechanisms in the offspring, thereby changing the propensity to obesity in adult life (McMillen et al., 2005; Martin-Gronert and Ozanne, 2006). However, evidence is increasing that the different outcomes of these adaptations on later health depend on the type, duration, period, and severity of the restriction and that they are, at least in part, gender specific.

Barker and collaborators in 1991 proposed the foetal origin of adult disease hypothesis (Barker and Osmond, 1986; Godfrey and Barker, 2001; Hales and Barker, 2001), This suggests that poor gestational nutrition leads to metabolic adaptations increasing not only the survival of the foetus but also the propensity to obesity and related alterations in adulthood, particularly under an obesogenic environment (Hales and Barker, 2001; Wells, 2007). Since then, considerable epidemiological evidence has been published showing an association between poor foetal growth and susceptibility to obesity and other diseases such as cardiovascular diseases, type 2 diabetes, and osteoporosis (Martorell et al., 2001; Langley-Evans, 2004). A number of animal studies have also related undernutrition during foetal life—due to maternal dietary restriction by calorie, protein or specific nutrient limitation—with lasting detrimental consequences on the homeostatic control of energy balance and obesity (Desai et al., 2007a; McMillen et al., 2008; Plagemann, 2008; Palou et al., 2010a).

On the other hand, unlike the number of studies found on foetal undernourishment, the evaluation of the long-term outcomes of early postnatal undernutrition during the suckling period, has received less attention. Studies carried out in humans have focused primarily on the consequences of energy restriction on the volume of milk production, but not on the potential effects on children's health (Dusdieker et al., 1994; Motil et al., 1995). In animal models, the positive or negative lasting effects of undernutrition during lactation are not clearly elucidated, as the conclusions are masked by the specific conditions tested: while severe food restriction during suckling—generally by means of increased litter size—has been related with growth retardation (Remmers et al., 2008a,b), moderate calorie or protein restrictions in dams during lactation have been associated with an improvement of insulin and leptin sensitivity in offspring later in life (Zambrano et al., 2006; Palou et al., 2010b).

**Figure 1:**
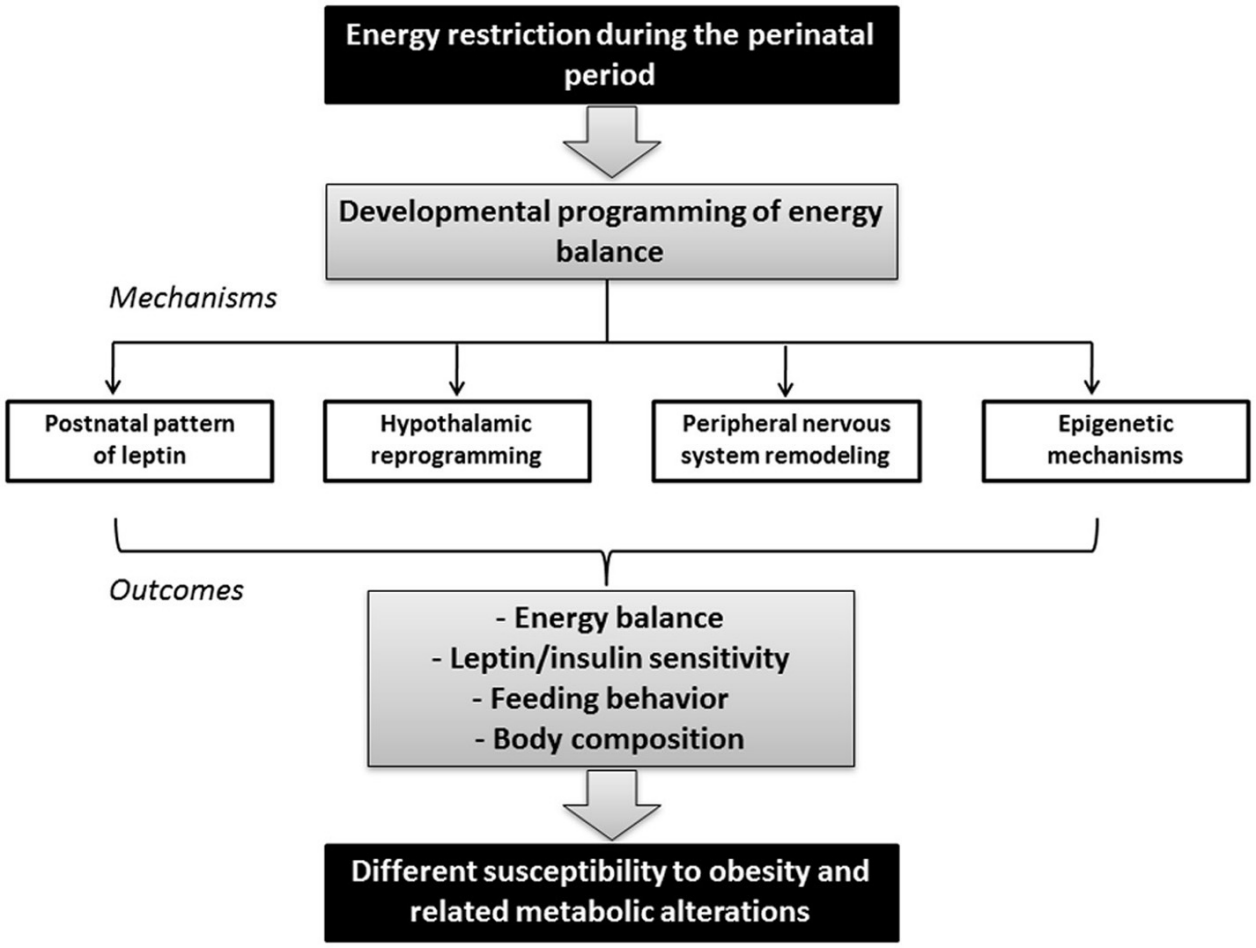
**Schematic diagram illustrating the link between maternal nutrition during the perinatal period and adult metabolic health.** Energy restriction during gestation and/or lactation may impact the developmental programming of energy balance in the offspring, depending on factors such as the type, duration, period, and severity of conditions. Mechanisms underlying these effects may include changes in the postnatal pattern of leptin, development of key structures such as the hypothalamus and the peripheral nervous system (which in turn may also be influenced by leptin), and epigenetic modifications. These changes in offspring may have long-term consequences in the susceptibility to obesity and other metabolic alterations by affecting the capacity to regulate energy balance, leptin and/or insulin sensitivity, feeding behavior and/or body composition.

All in all, there is increasing evidence of a close link between perinatal environment and adult metabolic health, affecting susceptibility to obesity and related metabolic alterations. Here we review the effects of maternal calorie restriction during gestation and lactation (see Figure 1) as well as the underlying mechanisms involved in the developmental programming of the processes controlling energy balance, by which a different propensity to metabolic disorders is early programmed, in order to identify prevention strategies from early stages of development.

## Programming effects of calorie restriction during gestation on subsequent body weight

The importance of maternal wellbeing on the future metabolic health of offspring is not new. There is strong evidence linking malnutrition during foetal life and a major propensity to acquire overweight later in life (Desai and Hales, 1997; Martin-Gronert and Ozanne, 2006). The Thrifty Phenotype hypothesis proposes that poor nutrition during early life leads to changes in tissue and organ functions, whereby the subject remains programmed to accumulate energy under a positive nutritional environment (Inoue et al., 2007; Wells, 2007; McMillen et al., 2008). Much evidence supports this idea, such as the emblematic example of the Dutch famine (Ravelli et al., 1976), where it was found that 19-year-old men conceived during the acute famine that devastated the western part of Holland during the last 6 months of World War II—whose mothers experienced poor nutrition in the first and second trimesters of their pregnancies—were more likely to be obese than their peers—whose mothers did not experience poor nutrition. Outcomes were different depending on the time of exposure. During the last trimester of pregnancy and the first months of life, exposure to energy restriction produced significantly lower obesity rates. Other epidemiological studies in humans have also related a low-birth weight to a greater risk of developing cardiovascular disease, hypertension, type 2 diabetes, and central obesity in adulthood (Barker et al., 1993; Martyn and Barker, 1994; Achard et al., 2006). In fact, human studies performed in the last decades suggest that 25–63% of adult diabetes, hypertension, and coronary heart disease could be attributed to low birth weight and/or to accelerated newborn-to-adolescent weight gain (Barker et al., 2002).

Nevertheless, most of the evidence associating foetal malnutrition and adult obesity are from animal models, particularly rats (Rasmussen, 1998). In this sense, severe maternal calorie restriction during pregnancy—up to 50%—which usually results in a reduced birth weight, has been associated with a greater incidence of obesity in adulthood, particularly when animals were exposed to an obesogenic environment (Thompson et al., 2007; McMillen et al., 2008). However, different outcomes have been described regarding subsequent body weight depending on the type, severity and timing of the restriction, and on the gender of the progeny (Symonds et al., 2004). Thus, male offspring of 50% calorie-restricted rats during the first 2 weeks of gestation displayed higher body weight and food intake than controls from the age of 5 weeks, whereas no significant differences were found in females (Jones and Friedman, 1982; Jones et al., 1984). On the other hand, Anguita and collaborators (Anguita et al., 1993)—using a comparable energy restriction treatment on pregnant rats—observed an impairment of normal weight in males and females despite no changes in food intake. However, the differences did not appear at the same age in both genders: whereas in males intrauterine malnutrition led to in impairment of normal weight gain and fat deposition from day 1 to day 53 of age, in females this resulted in pronounced fat accretion by the age of 53 days. In a different model of energy restriction, consisting of rat dams submitted to 50% food restriction from day 10 of pregnancy until delivery, pups showed intrauterine-growth restriction, but experienced very rapid catch-up growth after birth. Both males and females became obese at the age of 9 months (Desai et al., 2005). A more moderate gestational calorie restriction (30%) throughout gestation or during the first 2 weeks of pregnancy (Vickers et al., 2000, 2005) has also been related to hyperphagia, together with greater fat accumulation in offspring, particularly under a hyper-caloric diet, but without significant differences in body weight compared to controls. In addition, we have also described that lower calorie restriction (20%) during the first 12 days of gestation also has lasting effects in male and female offspring, programming both genders for increased food intake, but only resulting in greater body weight and body fat content in males (Palou et al., 2010a, 2012).

Thus, experimental studies in animals have contributed to providing a link between reduced birth weight in humans and subsequent health outcomes. However, the causes appear to be more complex than originally envisaged. Changes in maternal nutrition do not always result in altered foetal growth and can affect offspring health independently of size at birth (Harding et al., 2011). Moreover, maternal nutrition is important not only after conception. There is evidence showing that maternal undernutrition at distinct periods around conception has different effects on foetal growth. Periconception undernutrition in sheep has been shown to alter foetal metabolic and endocrine function and growth regulation and trajectory without intrauterine growth restriction (IUGR) (Rumball et al., 2009). Moreover, preconception undernutrition alone has been described to alter foetal growth responses to late gestation stressors (Rumball et al., 2009). These observations suggest that maternal nutrition is also important before conception.

In addition to maternal nutrition, other factors may also be of crucial importance concerning foetal development and subsequent health-related consequences. These factors include uterine and umbilical blood flows as well as placental transport and metabolism, since they may affect oxygen and nutrient supply to the foetus. Any interference in these steps may affect foetal nutrition and commonly results in growth restriction (Bloomfield and Harding, 1998). Different animal models of IUGR have been developed to investigate the mechanisms whereby this condition predisposes to subsequent development of metabolic diseases. These models are generally based upon uterine artery ligation, prenatal food or protein restriction, and exposure to specific hormones or to hypoxia during gestation. However, it must be pointed out that these conditions may differently influence later catch-up growth, which is believed to be a central component in the link between IUGR and the subsequent development of metabolic diseases in humans (Haugaard and Bauer, 2001; Shahkhalili et al., 2010).

Apart from the effects of overall maternal undernutrition during pregnancy, other studies have focused on the effects of foetal undernutrition by maternal protein restriction during gestation—generally performed by exposing dams to an isocaloric low protein diet (containing around 8% protein), compared with controls, fed 20% protein. Offspring born to protein restricted dams (8% protein) but suckled by normally fed dams were smaller at birth, but underwent rapid catch-up growth when suckled by normally fed mothers (Jennings et al., 1999). In adulthood these animals showed body weights similar to controls from 21 days of age, but had reduced longevity when fed *ad libitum* on standard chow diet. In another study, this very rapid catch-up postnatal growth after foetal protein restriction (8% protein) was also associated with increased body weight with respect to their controls at the age of 16 days (Coupe et al., 2010). In contrast, similar protein restriction (9% protein, compared with controls fed 18% protein) during pregnancy did not affect body weight of offspring of either males or females at the age of 1, 9, or 18 months; however, in senescence, these animals developed a metabolic syndrome-like phenotype (Erhuma et al., 2007). Neither were significant differences in body weight found in female offspring of moderate protein-restricted rats (30%) during gestation at the age of 24 weeks, although they did exhibit significantly greater food intake than their controls (Ikenasio-Thorpe et al., 2007).

Central and peripheral resistance to insulin and/or leptin signaling have been proposed as important mechanisms responsible for the deregulation of energy homeostasis, which may lead to obesity (Levin and Dunn-Meynell, 2002; Lustig et al., 2004; Esteghamati et al., 2009; Palou et al., 2010a) (see below). On the other hand, adiponectin, which has been described as an important adipokine related with obesity and insulin sensitivity, may also play a role in the early programming mechanisms involved in the effects of gestational undernutrition, as it exerts important effects on carbohydrate metabolism, improving glucose metabolism by increasing insulin sensitivity (Berg et al., 2001; Yamauchi et al., 2001). Furthermore, adiponectin has also antiatherogenic and anti-inflammatory properties (Fantuzzi, 2005). Unlike leptin, its levels are negatively correlated with body weight and fat content (Arita et al., 1999). Reduced adiponectin levels have been described in children born small for gestational age, in relation with their predisposition to develop insulin resistance and atherosclerosis (Cianfarani et al., 2004). Moreover, adiponectin levels in IUGR children were particularly low in those who showed postnatal catch-up growth, compared with the levels in IUGR children who remained small during childhood (Sancakli et al., 2008). These data suggest that adiponectin insufficiency may also play a role in the metabolic abnormalities observed in IUGR children and adults. However, there is still limited information available, and controversial data showing normal or higher adiponectin concentrations in IUGR insulin-resistant children have also been described (Evagelidou et al., 2007), suggesting that other factors and conditions may be influencing this association (Briana and Malamitsi-Puchner, 2009).

Another important aspect in the control of body weight and feeding behavior is the preference for certain foods. This is particularly significant in humans, where the obesity state is generally associated with greater food intake and appetite together with a preference for highly caloric food (Rissanen et al., 2002). A descriptive human study comparing the dietary preference of monozygotic twins who were discordant for obesity showed that the obese twin had a greater preference for fatty foods than the lean co-twin (Rissanen et al., 2002). Hence, programming food preferences in favor of high or low calorie food would be very important in the prevention of obesity development, even more so when high-fat foods are widespread in developed countries. In this sense, nutritional changes during perinatal development have been described to lead to differential programming of eating behavior, affecting not only the appetite but also food preferences, and being related with a propensity to obesity later in life. For instance, 20% maternal calorie restriction during the first half of gestation changed the dietary preferences of male offspring in adulthood; these animals showed a greater preference for fat-rich food than their controls. This could explain, at least in part, their increased food intake, under high-fat diet conditions, and their greater body weight. In contrast, females did not show differences in their dietary preferences with respect to their controls, and no significant differences in body weight were observed (Palou et al., 2010a). Thus, changes in food preferences could be one of the mechanisms that might explain the greater impact of moderate calorie restriction during gestation on subsequent body weight in male offspring (see Table 1). On the other hand, both male and female offspring of 50% protein-restricted-fed pregnant rats were programmed for a specific preference for energy-dense foodstuffs and hyperphagia, which was also related with greater body weight and body fat content in adulthood (Bellinger et al., 2004).

**Table 1:** **Impact of different percentages of calorie restriction during gestation or lactation on male and female offspring**.

**Parameter**	**Outcome in offspring (sex; age; diet)**	**Percentage of restriction and period**	**References**
**MATERNAL CALORIE RESTRICTION DURING GESTATION**			
Body weight	↑ (males; from d 74 to m 6; NF and HF diet)	20%, first 12 d of gestation	Palou et al., 2010a, 2012
	↑ (females; 170 d; HF diet)	30%, first 2 w of gestation	Vickers et al., 2005
	↑ (males; 5 w; NF diet)	50%, first 2 w of gestation	Jones and Friedman, 1982; Jones et al., 1984
	↑ (males and females; 9 m; NF diet)	50%, from d 10 of pregnancy until delivery	Desai et al., 2005
	↑ (males and females; 260 d; HF diet)	50%, whole gestation	Thompson et al., 2007
Food-intake	↑ (males and females; from d 21 to m 6; NF and HF diet)	20%, first 12 d of gestation	Palou et al., 2010a, 2012
	↑ (males; from d 22 to d 125; NF and HF diet)	30%, whole gestation	Vickers et al., 2000
	↑ (females; 170 d; NF and HF diet)	30%, first 2 w of gestation	Vickers et al., 2005
	↑ (males; 5 w; NF diet)	50%, first 2 w of gestation	Jones and Friedman, 1982; Jones et al., 1984
Preference for highly caloric food	↑ (males; 3 m; NF diet)	20%, first 12 d of gestation	Palou et al., 2010a
**MATERNAL CALORIE RESTRICTION DURING LACTATION**			
Body weight	↓ (males and females; from d 5 to w 28; NF and HF diet)	30%, whole lactation	Palou et al., 2010b, 2011b
	↓ (males and females; from d 10 to d 21; NF diet)	67%, whole lactation	Boxwell et al., 1995
	↓ (females; 30 d and 90 d; NF diet)	50%, d 1–15 or d 15–30, or d 1–30 of lactation	Šefčíková and Mozeš, 2002
Food-intake	↓ (males and females; from d 21 to w 28; NF and HF diet)	30%, whole lactation	Palou et al., 2010b, 2011b
	↑ (females; 90 d; NF diet)	50%, d 1–15 or d 15–30, or d 1–30 of lactation	Šefčíková and Mozeš, 2002
Preference for highly caloric food	↓ (females; 24 w; HF diet)	30%, whole lactation	Palou et al., 2010b

Results are from animal studies. Sex, age, and diet for the outcome reported are specified in brackets. Abbreviations: d, day; w, week; m, month; NF, normal-fat; HF, high-fat.

Thus, the different studies mentioned above mostly show a greater propensity to develop altered energy metabolism in adult life, in particular overweight and/or hyperphagia, after undernourishment during foetal development. This enhanced propensity to develop obesity is particularly clear in offspring of overall calorie-restricted dams, particularly when they undergo rapid catch-up postnatal growth, and is also exacerbated when animals are exposed to an obesogenic diet in adulthood. Mechanisms underlying the deregulation of food intake and energy balance, due to perinatal nutrition, could be related with hypothalamic alterations (Garcia et al., 2010) and a lower capacity to respond to insulin and leptin signaling (Palou et al., 2012), which are further commented below.

## Programming effects of calorie restriction during lactation on subsequent body weight

Lactation is one of the most important stages of development. Given that milk is the only source of food until the start of independent feeding (Babicky et al., 1972; Fiorotto et al., 1991), maternal nutrition during this period may be pivotal in programming the metabolic health of offspring. In this sense, the identification of nutritional and environmental conditions in dams that affect milk composition becomes very important in the prevention of future diseases in offspring.

However, unlike the contribution of the foetal period, the contribution of the early postnatal environment has received less attention, beyond the known beneficial effects of breastfeeding compared with infant formula against later pathologies, including obesity and related metabolic alterations (Von Kries et al., 1999). In addition, unlike the known effects of calorie restriction during foetal life increasing the risk of developing obesity, the lasting consequences of maternal undernutrition during the suckling period are uncertain. In fact, studies in humans are scarce, principally due to ethical aspects (Dewey, 1998a,b), and have been mainly focused on the amount of milk production by mothers rather than on the consequences in their infants (Coward et al., 1984; Prentice et al., 1984; Dusdieker et al., 1994). Lovelady and collaborators (Lovelady et al., 2000) described that moderate weight loss of 0.5 Kg per week—by restriction to 500 Kcal less than the control group and with moderate exercise—between 4 and 14 weeks postpartum in overweight lactating women who are exclusively breast-feeding, did not affect the normal weight gain of infants. A previous study by McCrory and collaborators (McCrory et al., 1999) also showed that an intervention for 11 days with 35% caloric restriction combined with exercise or not was not harmful for lactating women. In this study, no significant changes were detected in the amount and energy output of milk, frequency of nursing or weight gain of infants.

Regarding animal studies on intervention during lactation, most of these were performed by maternal restriction of overall calories or proteins or by direct food restriction of pups by increasing litter size. Results show different outcomes depending on the type and severity of the restriction. On the one hand, severe maternal calorie restriction (67%) throughout the lactation period (Boxwell et al., 1995) was shown to result in lower body weight of pups at weaning. Vicente and collaborators (Vicente et al., 2004) also showed that offspring of protein restricted dams (65% reduction) or energy restricted dams (to the same amount of calories eaten by the protein restricted group) during lactation had lower body weight at weaning. In adulthood the animals whose dams were fed the protein restricted diet weighed significantly less than the controls did. However, the animals whose dams were fed the energy restricted diet weighed more than the controls. Another study showed that female offspring of 50% calorie-restricted dams during the suckling period, although presenting a lower body weight than their controls, displayed increased food intake (Šefčíková and Mozeš, 2002). These conditions of undernutrition during the suckling period also affected feeding behavior in adulthood. Evaluation of feeding behavior under different experimental conditions revealed that these rats consumed significantly less food in a new (unfamiliar) environment than in the home environment (Šefčíková and Mozeš, 2002).

Comparable results to severe maternal restriction have been obtained by increasing litter size during lactation. Remmers and collaborators (Remmers et al., 2008a) showed that an increase of litter size from 10 to 20 pups per dam resulted in lower body weight and food intake of both males and female restricted pups in adulthood. However, these animals displayed reduced body dimensions at weaning and also showed incomplete catch-up growth size afterwards (Remmers et al., 2008a). Velkoska and collaborators (Velkoska et al., 2008) also demonstrated that pups raised in large litters (18 pups per litter) were lighter than their controls and remained significantly lighter throughout the study, with no evidence of catch-up growth.

On the other hand, unlike severe maternal food restriction during the suckling period, moderate maternal calorie restriction (30%) was described to result in lower body weight and fat content in both male and female offspring, but not to affect animals' body length (Palou et al., 2010b). The lower body weight was also associated to lower food intake, which was maintained from weaning to adulthood (Palou et al., 2010b). Moreover, these rats also showed a better response to high-fat diet and were protected against the increased body weight and hyperphagia exhibited by their controls under these dietary conditions (Palou et al., 2011b). Thus, these results suggest that moderate maternal calorie restriction during lactation protects against diet-induced obesity in rats, but does not impede catching up to normal body length and weight under standard diet conditions (Palou et al., 2010b, 2011b). Interestingly enough, these conditions of moderate calorie restriction during lactation also resulted in a different programming of food preferences; that is, female offspring of calorie-restricted lactating dams exhibited a lower preference for fat-rich food when exposed to high-fat diet feeding in comparison with their controls (Palou et al., 2010b).

The malnutrition produced by protein restriction of dams during the suckling period also seems to be related with a lower body weight of offspring, which is maintained in later life (De Souza Caldeira Filho and Moura, 2000; De Moura et al., 2007; Fagundes et al., 2007). However, there is controversial evidence regarding programming effects on food intake. For instance, 65% maternal protein restriction during the suckling period resulted in pups with lower food intake from weaning until the age of 53 days, but from that age onwards no significant differences were found (Teixeira et al., 2002; De Moura et al., 2007). In a similar experimental design, pups of protein-restricted dams during lactation did not eat less food than their controls when referred to body weight (Fagundes et al., 2007). On the other hand, offspring of protein-free nourished dams during suckling—when fed with a normal protein diet at weaning—did not show lower food intake compared to controls. Interestingly, when they were exposed to a protein-free diet in adulthood, the reduction in food intake was more marked than controls (Moura et al., 2002). In another study, both male and female offspring of 50% protein-restricted dams during suckling showed lower body weight and also lower total food intake at 100 days of age (Zambrano et al., 2006). Notably, and contrary to what is described concerning protein restriction during gestation, it is remarkable that reduced growth during lactation due to maternal protein restriction results in a permanent reduction in size and an increase in longevity (Jennings et al., 1999). It is not known whether the effects on longevity are directly related with particular features of lactation or with growth reduction. Lim and collaborators (Lim et al., 2006) also described that 56.5% maternal protein restriction during both gestation and lactation led to lower body weight of offspring which persisted into adulthood. However, body weight of offspring of 60% protein-restricted dams during pregnancy and lactation remained lower than controls until the fifth week, but later on differences in body weight between groups disappeared (Coupe et al., 2009b). These rats also displayed hyperphagia with respect to their controls (Coupe et al., 2009b).

All in all, animal studies show that dietary restriction during suckling by maternal restriction or by increasing litter size results in a different programming of subsequent body weight and food intake, which may change propensity to obesity. Thus, although moderate restrictions may have protective effects against overweight and improve metabolic health of offspring (Palou et al., 2010b, 2011b), severe deprivation may lead to detrimental effects on the normal development of animals (Boxwell et al., 1995; Šefčíková and Mozeš, 2002; Remmers et al., 2008a) (see Table 1). However, it must be stressed that these conclusions are based on animal studies, since there are no clear data available from humans. Therefore, we should be cautious in extrapolating these results to humans due to differences between species, particularly in the different timing of birth as regards organ maturity and metabolic development.

## Mechanisms linking perinatal nutrition and risk of obesity

The mechanisms underpinning maternal nutrition and programming of obesity risk in offspring are not clearly elucidated. One potential mechanism could be through permanent structural changes in key organs. In fact there are several examples in animal models showing lasting effects of the perinatal environment on brain development (Morgan and Naismith, 1982; Plagemann et al., 2000a; Garcia et al., 2010). Circulating hormones influence multiple aspects of hypothalamic development and play a role in directing formation of neural circuits. Thus, a deficiency in some of these essential factors during a critical period of development will have permanent structural consequences. Among these, leptin has been proposed as an important neurotropic factor during postnatal development (Bouret et al., 2004; Yura et al., 2005). Its deficiency during this period may have later consequences in body weight control (Palou and Pico, 2009; Pico et al., 2011). However, the brain is not the only tissue that is sensitive to perinatal conditions and responsible for the perinatal programming of body weight control. Recent data indicate that the development of other structures, such as peripheral innervations of the sympathetic nervous system (SNS), may also be altered during development and have an important impact on future adiposity and metabolism of the offspring (Garcia et al., 2011).

An alternative mechanism by which environmental factors at critical periods of development could have long-term phenotypic consequences may involve epigenetic modifications (Heijmans et al., 2009). In this section, recent progress in these fields is summarized (see Figure 1).

### Permanent structural changes in key organs during critical periods of development

#### Hypothalamic reprogramming in offspring by maternal under-nutrition

The hypothalamus is the centre that integrates peripheral and central signals to regulate metabolic status via the modulation of expression of energy-regulating peptides (Schwartz et al., 2000). Thus, permanent changes produced in the hypothalamus during critical periods of development could explain an impaired capacity to regulate energy homeostasis in adulthood. Alteration of the neuronal organization in the hypothalamus—either through nutrient restriction or through altered hormonal signaling in foetal life—may be an important mechanism underlying nutritional programming (Remmers and Delemarre-Van De Waal, 2011).

The hypothalamus consists of several nuclei; arcuate nucleus (ARC), paraventricular nucleus (PVN), dorsomedial hypothalamus (DMH), ventromedial hypothalamus (VMH), and lateral hypothalamic areas (LHA). The ARC contains orexigenic neurons that co-express neuropeptide Y (NPY) and agouti-related peptide (AgRP) and anorexigenic neurons that co-express pro-opiomelanocortin (POMC), a precursor of α-melanocyte-stimulating hormone (MSH), and cocaine-related and amphetamine-regulated transcript (CART). Both populations of neurons have receptors for peripheral signals, including those for insulin, ghrelin, and leptin. Nutritional programming of energy balance systems seems to involve adjustments in these areas at different levels: size of the areas and number of neurons found in each area, the interconnection that the neurons form between the different areas, the types of neurons present in each specific area and the expression of receptors for hormones and neuropeptides in these neurons (Plagemann et al., 2000a; Delahaye et al., 2008; Coupe et al., 2009a, 2010; Garcia et al., 2010) (see Table 2).

**Table 2:** **Hypothalamic reprogramming by maternal under-nutrition**.

**Type of alteration**	**Outcome in offspring (sex; age)**	**Type of maternal under-nutrition (period of exposure)**	**References**
Size of specific HT areas	↑ relative volume of VMH and ↓ absolute volume of PVN (males; 20 d)	8% low protein diet (G and L)	Plagemann et al., 2000a
Number of neurons	↑ density of neurons in PVN and VMH (males; 20 d)	8% low protein diet (G and L)	Plagemann et al., 2000a
	↓ neurons in ARC (males and females; 25 d)	20% caloric restriction (first 12 d of G)	Garcia et al., 2010
	↑ cell proliferation in PVN, VMH, and ARC (males; 8 d and 15 d)	50% caloric restriction (from d 14 of G and L)	Coupe et al., 2009a
Neuron connections	↓ αMSH fibers in PVN (males; 16 d)	8% low protein diet (G and L)	Coupe et al., 2010
	↓ POMC neuron fiber projections from ARC to the PVN (males; 21 d)	50% caloric restriction (from d 14 of G and L)	Delahaye et al., 2008
Types of neurons and expression of neuropeptides/receptors	↓ NPY positive cells in ARC		
	(males; 20 d)	8% low protein diet (G and L)	Plagemann et al., 2000a
	(males and females; 25 d)	20% caloric restriction (first 12 d of G)	Garcia et al., 2010
	↓ expression of POMC in ARC (males; 14, 17, 21, and 30 d)	50% caloric restriction (from d 14 of G and L)	Delahaye et al., 2008
	↓ expression of POMC, AgRP, and CART in hypothalamus (males and females; 25 d)		
	↓ expression of NPY in hypothalamus (females; 25 d)	20% caloric restriction (first 12 d of G)	Garcia et al., 2010
	↑ expression of NPY and AgRP in hypothalamus		
	(males; 22 d)	8% low protein diet (G and L)	Coupe et al., 2009b
	↑ NPY levels in PVN and LHA (males; 20 d)	8% low protein diet (G and L)	Plagemann et al., 2000b
	↓ expression of leptin receptor in hypothalamus		
	(males; 21 d)	50% caloric restriction (from d 10 of G and L)	Desai et al., 2007a
	(males and females; 25 d)	20% caloric restriction (first 12 d of G)	Garcia et al., 2010
	(males; 4 m)	30% caloric restriction (G)	Breton et al., 2009
	(females; 6 m)	20% caloric restriction (first 12 d of G)	Palou et al., 2012
	↓ expression of insulin receptor in hypothalamus		
	(males and females; 25 d)	20% caloric restriction (first 12 d of G)	Garcia et al., 2010

Variety of alterations described in the hypothalamus of offspring from mothers submitted to different types of energy restriction. The specific outcome is summarized including the sex and age in which the outcome has been reported. Abbreviations: AgRP, agouti-related peptide; ARC, arcuate nucleus; CART, cocaine-related and amphetamine-regulated transcript; d, day; G, gestation; HT, hypothalamus; L, Lactation; LHA, lateral hypothalamic areas; MSH, α-melanocyte-stimulating hormone; m, month; POMC, pro-opiomelanocortin; PVN, paraventricular nucleus; VMH, ventromedial hypothalamus.

Animal studies have shown that offspring of malnourished dams (both calorie and protein restrictions) that display obesity and/or metabolic alterations in adulthood showed alterations in the development and function of the hypothalamic pathways and systems involved in the regulation of energy homeostasis at weaning. In this sense, moderate calorie restriction (20%) during the first 12 days of gestation decreased the number of neurons in the ARC of offspring, accompanied by lower NPY positive neurons in this area (Garcia et al., 2010). Similarly, offspring of dams fed a low-protein diet during gestation and lactation showed a reduction of NPY neurons in the ARC as well as a greater relative volume of VMH and a lower absolute volume of the PVN (Plagemann et al., 2000a). In addition, in a similar model of protein restriction in dams, alterations in the establishment of neuron connections in pups' hypothalamus were observed in 12-day-old pups—as evidenced by low expression levels of genes involved in cell adhesion, microtubule organization, and axon elongation (Coupe et al., 2010). More severe food restriction (50% calorie restriction during pregnancy and lactation) resulted in an increase in cell proliferation in PVN, VMH, and ARC hypothalamic areas of pups (Coupe et al., 2009a), as well as reduced POMC neuron fiber projections from the ARC to the PVN (Delahaye et al., 2008). In fact, the anorexigenic pathway of melanocortins seems to be more sensitive to perturbations due to environmental conditions during early life, since a decreased number of positive αMSH neurons or altered αMSH fibers in the ARC and other nuclei have been observed in different maternal malnourished models (Delahaye et al., 2008; Breton et al., 2009; Coupe et al., 2010).

Interestingly, changes in hypothalamic structures were accompanied, in most cases, by adjustments in the expression of neuropeptides and by an altered response to fasting conditions at weaning (Cripps et al., 2005; Garcia et al., 2010), and also in adulthood (Ikenasio-Thorpe et al., 2007; Breton et al., 2009; Coupe et al., 2009b; Palou et al., 2012). In general, alterations in maternal nutrition that predispose offspring to obesity are accompanied by lower mRNA levels of the anorexigenic peptide POMC and, in some cases, CART in the ARC of offspring at weaning (Delahaye et al., 2008; Garcia et al., 2010). However, apparent incongruities in the levels of other neuropeptides were observed, depending on the animal model used. Moderate maternal calorie restriction during the first 12 days of gestation resulted in lower expression levels of NPY (in females) and AgRP (both sexes) in the hypothalamus at weaning (Garcia et al., 2010). However if mothers were subjected to protein restriction during gestation and lactation, their male offspring showed increased expression of these two orexigenic neuropeptides (Coupe et al., 2009b). In a similar model of maternal protein restriction, increased levels of NPY in the PVN and LHA areas have been described in 20-day-old pups (Plagemann et al., 2000b). No changes in the hypothalamic expression of NPY were observed in offspring of 50% calorie-restricted mothers during pregnancy and lactation (Delahaye et al., 2008). Although the effects of maternal under-nutrition during pregnancy depend on the type, quantity, and duration of the restriction, there are some similarities in the resulting effects on the hypothalamic pathways involved in food intake control, suggesting that common mechanisms may be in operation. The predominant result of these maternal nutritional conditions is a dominance of the orexigenic versus anorexigenic drive in the offspring, which are programmed for obesity and/or other metabolic alterations.

There is a clear association between leptin resistance and obesity (Ahima and Flier, 2000; Levin and Dunn-Meynell, 2002; Lustig et al., 2004). Leptin resistance programming may be one of the mechanisms that may account for the greater propensity to obesity. In fact, several studies have found an association between metabolic programming of obesity and the presence of lower expression levels of leptin (and generally also insulin) receptors in the hypothalamus of these animals, as well as altered signaling of these hormones (Desai et al., 2007a; Breton et al., 2009; Garcia et al., 2010; Palou et al., 2012). Offspring from 50% food-restricted dams showed reduced leptin-induced STAT3 phosphorylation at 1-day-old and impaired anorexigenic response to leptin, together with lower hypothalamic leptin receptor expression levels later in life (at 3 weeks of age) (Desai et al., 2007b). Lower levels of leptin and insulin receptors were observed in 25-day-old offspring of 20% calorie-restricted dams (Garcia et al., 2010; Palou et al., 2012). In addition, decreased expression levels of hypothalamic leptin receptor were also observed in adult offspring of calorie-restricted dams (Desai et al., 2007b; Palou et al., 2012), together with an altered response of leptin and insulin receptors to fasting conditions (Breton et al., 2009). Thus, the lower expression levels of these receptors together with the lower response to leptin and fed/fasting conditions may be one of the main mechanisms of nutritional programming of metabolic alterations.

Most of the studies regarding nutritional programming were performed only in males, but when both males and females were studied, sex-dependent differences were often found. The female offspring of calorie-restricted dams during the first part of pregnancy exhibited a more marked effect in the reduction in the number of cells in the ARC nucleus compared with the effect in male animals; female pups also showed decreased hypothalamic expression levels of NPY and POMC neuropeptides when no changes (in the case of NPY) or a lower effect (in the case of POMC) were observed in males (Garcia et al., 2010). In this sex-specific response to the nutritional environment, sex hormones seem to play an important role. In fact, when sex hormones were absent by gonadectomy, male animals with ventromedial hypothalamic lesions gained twice as much weight compared to males with gonads, but modest effects were observed in females (Kemnitz et al., 1977). Thus, as suggested (Anguita et al., 1993), the sex-hormone environment may have an important impact on the mechanisms whereby the systems that control energy balance can be altered by intrauterine malnutrition.

#### Peripheral nervous system remodeling in offspring of calorie-restricted dams

Apart from the effects on the central nervous system, there is also information on possible programming effects of perinatal conditions on the peripheral nervous system structures involved in the control of energy metabolism, specifically, in relation with the adipose tissue and gut innervations. Concerning adipose tissue, it is well known that sympathetic innervation is crucial in the regulation of body fat, lipid mobilization, and adipose tissue growth (Bartness and Bamshad, 1998). Thus, alterations in the development of SNS structures could involve alterations in the adiposity and the capacity of fat mobilization in adult animals.

The development of SNS innervation has been shown to be affected by nutritional disturbances during critical periods of development and to be responsible for some of the lasting effects of these conditions. Interestingly, these effects also seem to be sex-specific (Garcia et al., 2011). In this sense, reduced innervation in the inguinal adipose depot occurring as a consequence of prenatal under-nutrition was observed in male animals and was accompanied by increased size of this depot (due to increased fat cell proliferation and subsequent hypercellularity) in adult males exposed to a high-fat diet. None of these effects were observed in female animals. Thus, calorie restriction during gestation could lead to partial noradrenergic denervation of the inguinal adipose tissue of male animals and therefore favor the hyperplasia seen in this adipose depot in adulthood (Garcia et al., 2011).

Regarding gut innervations, long lasting effects of maternal undernutrition on the enteric SNS in offspring have also been described. Fifty percent maternal calorie restriction during the last 2 weeks of pregnancy has been shown to reduce enteric sympathetic innervations, thereby affecting the control of gut motility (Santer and Conboy, 1990), and also noradrenergic levels in the coeliac-superior mesenteric ganglion complex and the diameter of its neurons (Conboy et al., 1987). The latter effects were observed in both neonates and adult animals, thus demonstrating permanent effects of maternal under-nutrition on the development of sympathetic neurons.

#### Leptin as an essential factor during postnatal development

It is well established that circulating hormones represent important environmental signals and can act directly on the central nervous system to regulate its development and activity (Simerly, 2005; Bouret and Simerly, 2006). In particular, leptin has been shown to be an essential nutrient and a key metabolic regulator for developing pups when taken orally during the suckling period (Pico et al., 2007; Sanchez et al., 2008). The adipocyte-derived hormone leptin is known to play a crucial role in the central control of energy balance. However, recent findings indicate that neonatal leptin, instead of playing a main role in energy metabolism, is an important signal for the development of hypothalamic circuits controlling food intake and body weight, and this activity is restricted to a neonatal critical period that precedes leptin's acute regulation of food intake in adults (Bouret et al., 2004; Yura et al., 2005). A lack of leptin during early life in rodents compromises the neuronal organization of hypothalamic nuclei involved in food intake control, affecting sensitivity to this hormone in adulthood (Bouret et al., 2004; Yura et al., 2005; Bouret, 2009). In fact, leptin-deficient ob/ob mice present reduced AgRP and α-MSH fiber density in the PVN and this defect is reversed by chronic leptin injection during the first week of life (Bouret et al., 2004). Leptin is also required for normal neuronal and glial maturation in the mouse nervous system (Ahima et al., 1999), adult neuronal hippocampal neurogenesis (Garza et al., 2008), and dendrite formation (O'Malley et al., 2007). In addition, a neonatal leptin surge has been related to neuron differentiation and migration, whereas a low leptin level maintains neural progenitor cells (Udagawa et al., 2007). The precise way in which leptin exerts these effects, and the site of leptin action is unclear, although a molecular mechanism involving leptin receptor and ERK and STAT3 signaling has recently been postulated to be responsible for directing formation of NPY and POMC projections (Bouret et al., 2012).

In rodents, leptin levels are very low at birth, but several studies have described a surge of leptin around postnatal day 10–14 in the rat (Rayner et al., 1997; Ahima et al., 1998; Bautista et al., 2008), at a time when the hypothalamic circuits are immature. This has been correlated with maturation of the central nervous mechanisms that regulate appetite in later life (Bouret and Simerly, 2007; Metges, 2009; Pico et al., 2011). An alteration or disruption in this leptin surge has been related with long-lasting effects on the metabolism, including increased susceptibility to a postnatal obesogenic diet. In this sense, perinatal undernutrition, especially during foetal life, has been shown to result in a drastic reduction or even lack of plasma leptin surge in rats. This is accompanied by marked alterations of the ARC POMC system and impaired insulin and leptin sensitivity (Delahaye et al., 2008; Palou et al., 2012). In addition, a reduced, delayed normal leptin surge in offspring from dams fed an isocaloric low-protein diet during gestation (Bautista et al., 2008; Coupe et al., 2010) associated with intrauterine growth retardation has been described in rats. In mice, perinatal undernutrition has been associated with an earlier occurrence of the postnatal leptin surge (Yura et al., 2005); while a premature leptin surge induced by exogenous leptin administration in control offspring led to accelerated high-fat-diet-induced obesity (Yura et al., 2005). Therefore, both the magnitude and timing of the leptin surge appears to be important for the development of metabolic systems (Sullivan and Grove, 2010). Knowing the effect of leptin on the developmental programme of the hypothalamic pathways involved in energy homeostasis and the findings on disturbances in leptin surge during a critical time-window have shed new light on how maternal undernutrition contributes to programming further metabolic alterations in offspring (Martin-Gronert and Ozanne, 2006; Bouret, 2009; Metges, 2009; Sullivan and Grove, 2010). Importantly, injections of exogenous leptin in offspring of undernourished mothers during early postnatal life reverses the postnatal sequelae induced by developmental programming, including calorie intake, locomotor activity, body weight, fat mass, as well as hyperinsulinaemia and hyperleptinaemia (Vickers et al., 2005, 2008).

The origin of the postnatal surge of plasma leptin is still controversial. In contrast to adults, in whom leptin is mainly produced by the adipose tissue, the situation during the perinatal period is different, with the mother contributing to the supply of leptin for the foetus and newborn (Palou and Pico, 2009; Pico et al., 2011). Leptin is naturally present in breast milk (Casabiell et al., 1997; Houseknecht et al., 1997), and leptin supplied from maternal milk has been shown to be absorbed by the immature stomach of the neonate rat (Casabiell et al., 1997; Oliver et al., 2002; Sanchez et al., 2005) and transferred to the bloodstream (Casabiell et al., 1997; Sanchez et al., 2005). Therefore, during the suckling period, maternal milk may substantially contribute to circulating leptin in rats, at a time when the adipose tissue is still immature (Palou and Pico, 2009). This is supported by the fact that leptin concentrations rapidly decline to undetectable levels in neonate pups when separated from their mothers (Oliver et al., 2002).

Evidence for the essential role of leptin during the suckling period has been obtained from both animal and human studies. Studies in rats show that the intake of physiological doses of leptin during the suckling period prevents the animals from overweight and obesity and other metabolic alterations associated with feeding a high-fat diet (Pico et al., 2007; Sanchez et al., 2008; Priego et al., 2010). In addition, leptin treatment during lactation has lasting effects on the expression of the hypothalamic factors involved in the control of food intake, particularly POMC, leptin receptor, and suppressor of cytokine signaling 3 (SOCS3) (Pico et al., 2007). Thus, leptin may be important during the lactation period in both regulating neonate food intake and affecting the developmental events involved in the control of energy balance in adulthood (Palou and Pico, 2009). In addition, leptin during the suckling period has also been shown to programme a better response of the adipose tissue under high-fat diet conditions, by preventing the decrease of leptin receptor in internal depots and increasing the oxidative capacity of this tissue (Priego et al., 2010). Improvement of the peripheral action of leptin may be associated with a better handling and partitioning of excess fuel, increasing the sensitivity of these rats to insulin (Sanchez et al., 2008) and preventing other metabolic disorders related with high-fat diet feeding, such as hepatic lipid accumulation (Priego et al., 2010). Therefore, leptin may exert regulatory effects, not only at a central level, but also peripherally.

Indirect evidence of the role of breast milk leptin during lactation in humans has also been obtained (Miralles et al., 2006; Doneray et al., 2009; Schuster et al., 2011). Miralles and collaborators (Miralles et al., 2006) showed a negative correlation between milk leptin concentration and body weight gain of infants until 2 years of age. Doneray and collaborators (Doneray et al., 2009) also found a negative correlation between leptin concentration in mature milk and BMI increase of infants during the first month of life. Furthermore, Schuster and collaborators (Schuster et al., 2011) showed a negative association between breast milk leptin levels and infant weight gain over 6 months of lactation. Thus, moderate milk-borne maternal leptin appears to give moderate protection to infants from excess weight gain.

Leptin in milk comes from both the maternal blood source (Casabiell et al., 1997) and from its production in the mammary gland (Smith-Kirwin et al., 1998). Blood leptin seems to be differentially regulated during lactation. Ferreira and collaborators (Ferreira et al., 2007) showed that isocaloric protein-restricted rats during the first 14 days of lactation displayed higher serum leptin levels than their controls, despite having lower body weight. Increased leptin levels in 10-day-old offspring from rats submitted to a protein-free diet during lactation compared to offspring from control dams were also described (Moura et al., 2002). Interestingly, we have also reported that 30% calorie-restricted rats during lactation had, at weaning, higher mRNA and protein levels of leptin in mammary gland compared to their controls; despite exhibiting a reduction in the weight of mammary gland compared with controls, total leptin abundance estimated in the whole mammary gland was still higher than in their controls (Palou et al., 2010b). Whether these changes in leptin production by mammary gland, with the putative changes in leptin supply to the offspring, are responsible for some of the beneficial effects of moderate food restriction during lactation on the offspring needs further evaluation.

### Epigenetic mechanisms

Epigenetic refers to modifications of DNA and the DNA packaging proteins (histones) that regulate gene activity, including DNA methylation and post-translational modifications of histones (by methylation, acetylation, phosphorylation, and ubiquitination). Different epigenetic states on identical DNA sequences can lead to alternative gene expression levels. For instance, it is known that cytosine methylation within CpG dinucleotides of DNA acts in concert with other chromatin modifications to heritably maintain specific genomic regions in a transcriptionally silent state (Bird, 2002; Martin-Gronert and Ozanne, 2010).

In this line, the putative role of epigenetic modifications mediating the long-term-effects of early life nutrition is promising, and there are several examples that illustrate this. For instance, alterations in histone modifications have been found to be involved in mediating the effect of calorie restriction during the second half of pregnancy in repressed skeletal muscle GLUT4 transcription in rats (Raychaudhuri et al., 2008). Maternal protein restriction during gestation in rats has also been described to alter the methylation status of the promoters of POMC (Coupe et al., 2010), glucocorticoid receptor (Lillycrop et al., 2005), PPARα (Lillycrop et al., 2008), and angiotensin receptor (Bogdarina et al., 2007) genes. Maternal undernutrition during gestation and lactation with a low-protein diet in mice—which results in higher food intake in offspring but lower body weight in adulthood—has also been described to promote hypomethylation in the promoter of leptin, which is related with a stronger expression of this gene in response to food intake (Jousse et al., 2011).

In addition, other genes considered to be pivotal in the system that controls body weight appear to be related to processes that are likely to involve epigenetic modifications. This is the case of the FTO locus, which is a DNA-demethylase enzyme (Gerken et al., 2007); the MC4R gene, which has reduced methylation following long-term exposure to a high-fat diet (Widiker et al., 2010); and the PPARγ protein which interacts with histone acetyltransferases during adipogenesis (Choy et al., 2010). The effects of diet on methylation of POMC (Plagemann et al., 2009; Palou et al., 2011a), and on the methylation of the leptin gene (Milagro et al., 2009) are also suggestive.

The recent studies of Heijmans and collaborators demonstrate that prenatal environmental conditions are also associated with permanent changes in the human epigenome (Heijmans et al., 2009): individuals who were exposed to intrauterine famine during the Dutch Hunger Winter had altered methylation of the insulin-like growth factor 2 gene in white blood cells in adulthood (Heijmans et al., 2008). Some of the persistent changes in DNA methylation, described in prenatal famine exposure, seem to depend on the gender of the exposed individual and the gestational timing of the exposure (Tobi et al., 2009). Moreover, from association studies of periconceptional exposure to the Dutch Famine and genetic variation, it is shown that both environmental and genetic factors could have independent, additive, similarly sized effects on DNA methylation at the same regulatory site (Tobi et al., 2012).

On the other hand, and in accordance with the previously discussed role of leptin influencing different aspects of hypothalamic development during postnatal life, this hormone could also play a role in the establishment of DNA methylation patterns and its response to dietary conditions in later life (Palou et al., 2011a). In fact, differences in POMC promoter methylation have been found in rats treated with physiological doses of oral leptin during lactation after exposure to high-fat diet conditions. This pattern may contribute to explain differences occurring in the expression levels of POMC in hypothalamus, and the apparently improved capacity to regulate food intake, thus helping to protect animals against excess weight gain in adulthood (Palou et al., 2011a).

## Perspectives

Given the current obesity epidemic there is an urgent need for interventional strategies, even from early stages of life. In this sense, foetal and early postnatal time periods are important for determining future risk of obesity, type 2 diabetes and other features of the metabolic syndrome, regardless of genetic or further environmental factors. Animal and epidemiological studies provide clear evidence for the relationship between maternal nutrition—including not only over-nutrition, but also under-nutrition—and the risk of obesity in offspring, although with different programming effects depending on the magnitude of the dietary alteration and the specific period. Thus, they represent invaluable tools in the investigation of mechanisms underpinning this linkage as well as in providing a potential therapeutic target to stem the growing epidemic of obesity.

### Conflict of interest statement

The authors declare that the research was conducted in the absence of any commercial or financial relationships that could be construed as a potential conflict of interest.
